# In-depth analysis of large-scale screening of WRKY members based on genome-wide identification

**DOI:** 10.3389/fgene.2022.1104968

**Published:** 2023-01-09

**Authors:** Haoyu Pan, Yu Chen, Jingyi Zhao, Jie Huang, Nana Shu, Hui Deng, Cheng Song

**Affiliations:** ^1^ College of Biological and Pharmaceutical Engineering, West Anhui University, Luan, China; ^2^ School of Life Science, Anhui Agricultural University, Hefei, China

**Keywords:** computational analysis, bioinformatics, in-depth analysis, expression profile, WRKY transcription factor

## Abstract

With the rapid advancement of high-throughput sequencing technology, it is now possible to identify individual gene families from genomes on a large scale in order to study their functions. WRKY transcription factors are a key class of regulators that regulate plant growth and abiotic stresses. Here, a total of 74 *WRKY* genes were identified from *Dendrobium officinale* Kimura et Migo genome. Based on the genome-wide analysis, an in-depth analysis of gene structure and conserved motif was performed. The phylogenetic analysis indicated that DoWRKYs could be classified into three main groups: I, II, and III, with group II divided into five subgroups: II-a, II-b, II-c, II-d, and II-e. The sequence alignment indicated that these WRKY transcriptional factors contained a highly conserved WRKYGQK heptapeptide. The localization analysis of chromosomes showed that *WRKY* genes were irregularly distributed across several chromosomes of *D. officinale*. These genes comprised diverse patterns in both number and species, and there were certain distinguishing motifs among subfamilies. Moreover, the phylogenetic tree and chromosomal location results indicated that *DoWRKYs* may have undergone a widespread genome duplication event. Based on an evaluation of expression profiles, we proposed that DoWRKY5, 54, 57, 21, etc. may be involved in the transcriptional regulation of the JA signaling pathway. These results provide a scientific reference for the study of *DoWRKY* family genes.

## Introduction

External environment seriously affect plant growth and food security (Waqas et al., 2019). Among them, abiotic stresses, such as UV-B, drought, chilling, heavy metals, etc., inevitably lead to the decline of medicinal material quality and the accumulation of harmful substances (Jiang et al., 2020). The discovery of large-scale gene families that are involved in abiotic stress opens up new ways to deal with and control harmful conditions (Song et al., 2022a). Bioinformatics is an emerging field developed by integrating biology with computer science and mathematics (Kan et al., 2021). It tackles the barrier of processing huge amounts of biological data by applying computer science and technology and statistical methodologies thoroughly. With the advancement of high-throughput sequencing technology, genome assembly and sequencing, in conjunction with bioinformatics algorithms, have catapulted life sciences into the “omics” era (Jing and Liu, 2018; Song et al., 2021). The quantity of data contained in these omics metadata is enormous, and the relationships between the datasets are intricate. To comprehend the relationship between the structure and function of regulatory elements, it is often necessary to study several genes simultaneously (Patro et al., 2017). This mainly includes structural genomes, comparative genomes, and functional genomes. The investigation of the differentially expressed genes generated from the experiment involves large-scale data analysis of online services and local area networks, providing researchers with reference and optional data analysis tools (Kang et al., 2020). Computational biology is frequently employed to decode medicinal plant genomes, pan-genomes and molecular markers, as well as to explore the origin and evolution of related species, structural variation of genomes, neofunctionalization of gene families, and systematic biology, among other applications (Nadler et al., 1997). In terms of genome assembly, the assembly strategy and an algorithm based on overlapping graphs are applied to construct the process. PacBio CCS sequencing with Hi-C technology, Oxford Nanopore, and other third-generation sequencing technologies has been extensively employed to sequence non-model species in recent years (Sun et al., 2020; Ye et al., 2021). In terms of gene prediction, *de novo* sequencing based on hidden Markov models enhances prediction accuracy (Wang F et al., 2021). Regarding functional gene mining, bioinformatics can closely integrate the metabolome, genome, transcriptome and proteome, and examine the natural variation of metabolic regulatory genes using principle component analysis, hierarchical clustering analysis, and correlation analysis (Srivastava et al., 2018). Some researchers also use metabolome-genome wide association analysis (mGWAS) to investigate the underlying genetic basis of metabolite biosynthesis pathways (Zhang et al., 2020).

*D. officinale* is a perennial herb in the orchid family that belongs to the Dendrobium genus (Wang Y et al., 2021). It is mainly distributed in the Ta-pieh Mountain of Anhui, Zhengjiang, Guangzhou, Guangxi, Yunnan, and other provinces (Song et al., 2022b). *D. officinale* has considerable effects on immunity and hematopoiesis, as well as anti-oxidation, anti-tumor, anti-fatigue, and hypoglycemic properties (Song et al., 2022d). WRKY transcription factor family is one of the most extensive transcription factor families found in higher plants (Li et al., 2020). It is a crucial class of transcription factors that regulates plant growth, development, and stress tolerance (Guo et al., 2019; Jue et al., 2018). The representative aspect of WRKY transcription factors is the conserved WRKY domain, which consists of the highly conserved WRKYGQK heptapeptide and C2-H2 (C-X4-C-X22-23-H-X1-H) or C2-HC (C-X4-C-X23-H-X1-C) type zinc finger motifs (Cui et al., 2018; Jiao et al., 2018). WRKY transcription factors have a strong affinity for the common W-box element, which has a structural base consisting of a heptapeptide sequence and a zinc finger motif (Nan and Gao, 2019). Accordingly, WRKY proteins are classified into three major groups (I-III) based on the number of WRKY domains and the structure of their zinc finger motifs. The group II is further divided into five subclasses: II-a, II-b, II-c, II-d, and II-e. Since the first WRKY gene SPF1, was cloned from sweet potato, a vast number of WRKY members have been discovered in plants (He et al., 2017; Hu et al., 2021).

Genome-wide identification of gene families clarified the structure and function of each gene, and provided a theoretical foundation for future functional verification (Tang et al., 2021). Simultaneously, using the homologous gene alignment of many species, related gene sequences with high affinity for their motifs may be determined, as they are regarded as the probable functions of homologous genes. In 2015, Chen and his collaborators started developing BioCJava (the origin of TBtools). It is a biological large-scale data analysis software written in Java with over 140 functional modules and a good human-computer interface. The JIGplot engine provides a user-friendly interface for displaying graphs (Chen et al., 2020). Here, bioinformatics methods were used to identify the DoWRKY transcription factors. The amino acid alignment, gene structure analysis, phylogenetic tree construction, and chromosomal location were performed by TBtools (v.1.097), MEGA (v.6.06), and some other bioinformatics analysis softwares. The conserved motifs, *cis*-acting regions, and amino acid binding site found in the data can help enrich our understanding of WRKY genes and roles, as well as provide some theoretical references for further investigation.

## Materials and methods

### Identification of the *WRKY* genes in *D. officinale*

The latest *D. officinale* genome and annotation files were obtained from the NCBI genome database (BioProject accession: PRJNA662181). The AtWRKY sequence files and seed files (PF03106) from the Arabidopsis Information Resource (https://www.arabidopsis.org) and Pfam (http://pfam.xfam.org/) web services allow us to download the hmm file of the hidden Markov model containing the WRKY typical conserved domains (Supplementary Table S1). MEGA, and TBtools software are used for further sequence analysis (Tamura et al., 2013; Chen et al., 2020). The hmmbuild command in the hmmer package was used to create the WRKY.hmm file from the seed file. Following the acquisition of the WRKY.hmm file, the hmmsearch command was used to retrieve the WRKY.out file. To verify the comparison results, the TBtools software was used to extract the protein sequence of the WRKY proteins from the.out file to obtain the WRKY.fas file, resulting in a screened sequence file only containing the target protein. These proteins were submitted to the batch sequence search of pfam for verification. Subsequently, all candidate *DoWRKYs* were verified using Pfam and SMART to confirm that they contained the core domains. Based on the sequence alignments generated by the ClustalX software (http://www.clustal.org/clustal2/), all potentially redundant WRKY sequences were discarded. A preprint has previously been published (Pan et al., 2022).

### The phylogenetic analysis and amino acid alignment of WRKY transcription factors

MEGA 6 (v.6.06) was used to create a protein sequence alignment project (Tamura et al., 2013). The screened DoWRKYs and the AtWRKYs were concurrently imported to construct an alignment. After the alignment, the sequence differences of the WRKY transcription factors of the two species are retrieved, and the genetic relationship between the different WRKY transcription factors of the two species can be detected. The phylogenetic tree was constructed based on the WRKY conserved domain of *D. officinale* and *A. thaliana* using a neighbour-joining method (execution parameters: Poission correction, pairwise deletion, and bootstrap of 1,000 repetitions). The related DoWRKY proteins can be classified by referring to the classification of defined AtWRKY proteins. The IQ-TREE software (v.1.6.12) was used to further determine the phylogeny relationship between DoWRKYs and AtWRKYs (Nguyen et al., 2014). Our best-fit model to determine sequence alignment through method optimization is VT + R7. Using this model, we construct the phylogenetic tree based on the maximum likelihood method. iqtree.exe -s./bidui.fas-m VT + R7-bb 1000-alrt 1000-nt AUTO was the running parameter. To analyze the amino acid sequence of DoWRKYs and determine the conserved domains, the GeneDoc (v. 2.7) (https://genedoc.software.informer.com/2.7/) was used. The phylogenetic trees were used to assess the homology of DoWRKYs with other WRKY proteins.

### The conserved motif analysis of *WRKY* transcription factors

The screened DoWRKY protein sequence.fa file was submitted to the MEME online web service (http://meme.nbcr.net/meme/tools/meme) for searching the conserved motif. The following settings were set: The number of motifs is set to 20. The width of the advanced option motifs was in the range of 6–200. All of the other parameters were set to default. The xml file can be obtained after submitting it to the MEME-suite service (https://meme-suite.org/meme/tools/meme), or by utilizing the MEME suite Wrpper from the TBtools package (Chen et al., 2020). To obtain a map of conserved motif analysis, the biosequence structures illustrator was used to display a motif pattern with the default settings.

### The gene structure analysis and chromosomal localization of *WRKY* transcription factors

Using the biosequence structures illustrator tool, the gene structure of *DoWRKYs* was obtained based on the gene annotation file. The settings are made in order to obtain the generic structural map. The color and height of the gene structure of CDS and UTR regions can be adjusted. Using the genes on chromosomes function module of TBtools, the gene location was visualized based on the gene annotation file. All of the *WRKY* genes on the pseudochromosomes can be found by putting in the file after the gene names have been changed.

### The expression profile of *DoWRKY* genes under different MeJA treatment

The expression pattern of WRKY gene family was further analyzed using the transcriptome data of *D. officinale*. First of all, the clean data were mapped to the latest genome of *D. officinale* (https://www.ncbi.nlm.nih.gov/genome/31795?genome_assembly_id = 1672529). Then, the HISAT2 software was applied to align the obtained clean data with the reference genome, and the comparison efficiency was calculated to evaluate the assembly quality of the selected reference genome (Pertea et al., 2016). The StringTie software was used to assemble the aligned reads, and the obtained unigenes were quantitatively analyzed (Pertea et al., 2015). The gene expression levels of *DoWRKY* genes were represented using the fragments per kilobase of transcript per million mapped reads (FPKM) method (Sims et al., 2014). TBtools software was used to build a heatmap illustrating the expression profile of groups with drastically altered genes (Chen et al., 2020).

## Results

### Identification and category of *DoWRKY* genes

**FIGURE 1:**
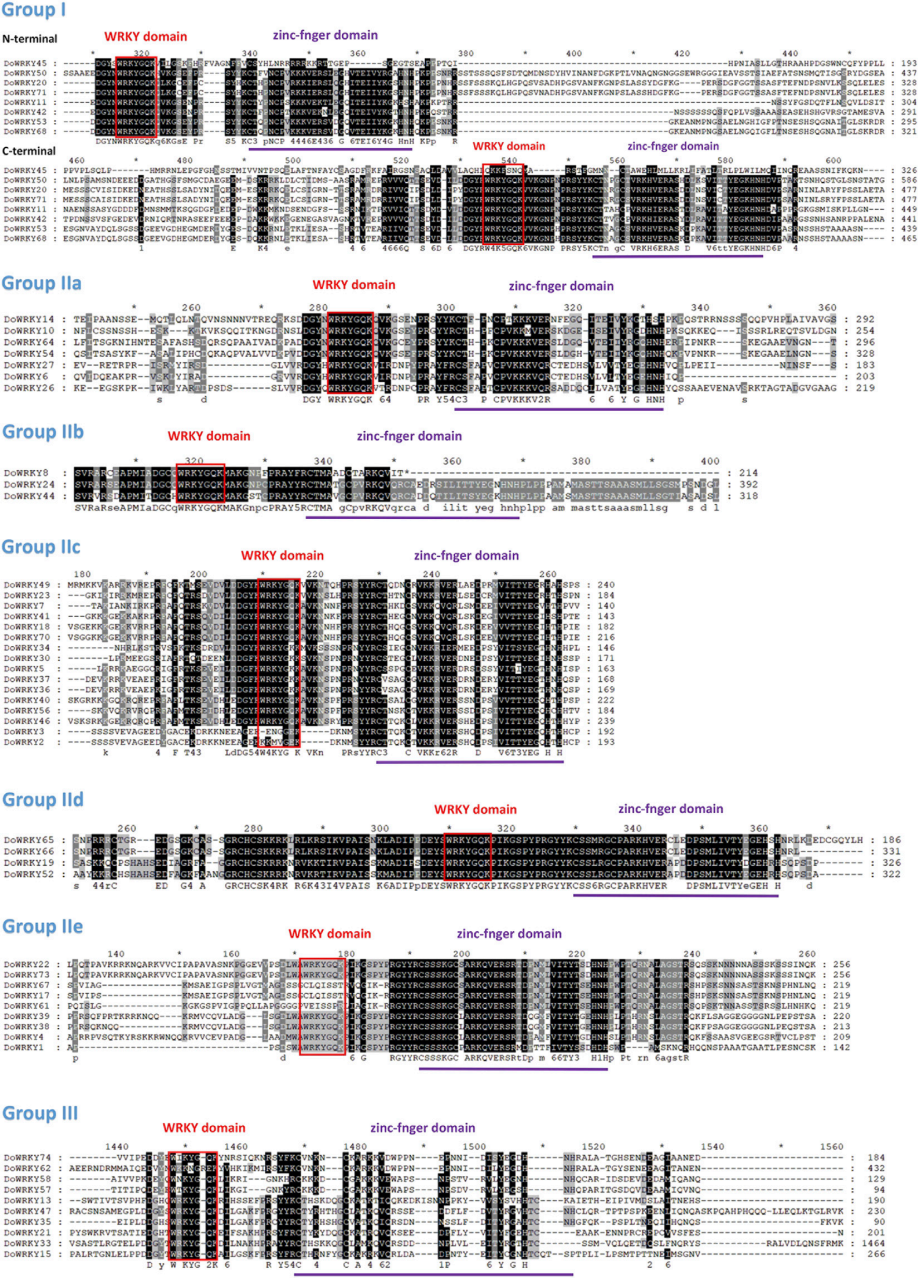
Multiple sequence alignment of the WRKY domain among DoWRKY proteins. Red indicates conserved WRKY domains. Purple indicates zinc finger motifs and dashes indicate gaps.

To create the WRKY.hmm file from the seed file, the hmmbuild function of the hmmer software was employed. Using the hmmsearch function, the WRKY.out file was retrieved based on the WRKY.hmm file. TBtools software was used to extract the protein sequence of the WRKY protein id in the initial alignment of the.out file, obtain the WRKY transcription factor sequence file, submit the DoWRKY protein sequence file to the Pfam website for verification, and then compare the NCBI database to remove those that do not meet the requirements (Table 1). We renamed *DoWRKY1*-*DoWRKY74* after the analysis of chromosomal location. The basic properties of these genes include subfamily classification, type of zinc finger structure, number of amino acids, molecular weight, and isoelectric point. The full name of the protein spans from 72 to 1,583 aa (DoWRKY60 and DoWRKY33). Some truncated proteins may be alternative splices of their paralogs, such as DoWRKY60, DoWRKY9, and DoWRKY31. DoWRKY33 has longer amino acids, suggesting that it may contain LTR-retrotransposons. It was indicated that DoWRKY transcription factors could be classified into three types: group I, group II, and group III, with group II further subdivided into five subclasses: II-a, II-b, II-c, II-d, and II-e (Figure 1). Both groups I and II have two highly conserved WRKY amino acid sequences. Group II has a WRKY amino acid sequence. Both groups I and II have a C2-H2 type of zinc finger structure, and group III has a WRKY amino acid sequence. There are 13 class I WRKY proteins, eight class II-a WRKY proteins, three class II-b WRKY proteins, 16 class II-c WRKY proteins, and six class d WRKY proteins, 11 class II-e WRKY proteins, 17 class III WRKY proteins.

**TABLE 1:** Classification and structural properties of *DoWRKYs*.

Gene ID	Name	Category	Zinc-finger type	Length (aa)	MW (Da)	pI
Dof016042	*DoWRKY45*	Group I	C2—H2	380	42682.22	9.83
Dof001913	*DoWRKY9*		C2—H2	93	10175.46	8.80
Dof019768	*DoWRKY60*		C2—H2	72	8230.16	5.03
Dof016991	*DoWRKY50*		C2—H2	672	73250.54	5.22
Dof017003	*DoWRKY51*		C2—H2	562	61265.13	5.36
Dof026529	*DoWRKY72*		C2—H2	509	55013.15	5.03
Dof006797	*DoWRKY25*		C2—H2	639	69607.09	5.33
Dof005813	*DoWRKY20*		C2—H2	577	64455.97	6.30
Dof026398	*DoWRKY71*		C2—H2	571	64470.98	6.33
Dof002660	*DoWRKY11*		C2—H2	509	56851.54	7.73
Dof014177	*DoWRKY42*		C2—H2	542	59950.78	8.06
Dof017739	*DoWRKY53*		C2—H2	472	51704.53	8.41
Dof025950	*DoWRKY68*		C2—H2	498	54579.63	7.30
Dof003184	*DoWRKY14*	Group IIa	C2—H2	427	46711.25	8.19
Dof002619	*DoWRKY10*		C2—H2	425	47642.88	8.92
Dof024906	*DoWRKY64*		C2—H2	367	39655.75	5.58
Dof018793	*DoWRKY54*		C2—H2	412	43363.11	6.20
Dof006875	*DoWRKY27*		C2—H2	222	26032.50	8.50
Dof001298	*DoWRKY6*		C2—H2	220	25160.72	9.26
Dof009263	*DoWRKY32*		C2—H2	191	21028.51	5.53
Dof006874	*DoWRKY26*		C2—H2	277	29958.63	8.09
Dof001711	*DoWRKY8*	Group IIb	C2—H2	214	24033.43	9.75
Dof006708	*DoWRKY24*		C2—H2	555	59518.67	6.48
Dof014830	*DoWRKY44*		C2—H2	451	48448.56	5.43
Dof016482	*DoWRKY49*	Group IIc	C2—H2	262	29674.42	9.42
Dof006535	*DoWRKY23*		C2—H2	198	22657.29	8.80
Dof001611	*DoWRKY7*		C2—H2	158	18462.59	9.02
Dof013755	*DoWRKY41*		C2—H2	162	18888.39	9.67
Dof004579	*DoWRKY18*		C2—H2	236	27312.74	8.93
Dof026259	*DoWRKY70*		C2—H2	236	27020.00	7.72
Dof010331	*DoWRKY34*		C2—H2	154	17889.66	5.57
Dof008649	*DoWRKY30*		C2—H2	193	21414.66	4.85
Dof000608	*DoWRKY5*		C2—H2	187	20957.22	6.08
Dof011349	*DoWRKY36*		C2—H2	311	35396.81	5.54
Dof011357	*DoWRKY37*		C2—H2	230	26124.26	5.31
Dof013129	*DoWRKY40*		C2—H2	317	34590.53	5.59
Dof019154	*DoWRKY56*		C2—H2	255	27854.72	6.24
Dof016156	*DoWRKY46*		C2—H2	298	33662.19	6.19
Dof000419	*DoWRKY2*		C2—H2	313	33980.01	6.37
Dof000427	*DoWRKY3*		C2—H2	272	29356.20	5.02
Dof005134	*DoWRKY19*	Group IId	C2—H2	336	35975.83	9.82
Dof017607	*DoWRKY52*		C2—H2	331	35453.51	9.84
Dof003967	*DoWRKY16*		C2—H2	320	36264.86	5.81
Dof019027	*DoWRKY55*		C2—H2	293	33040.38	5.34
Dof025067	*DoWRKY65*		C2—H2	281	31418.80	9.89
Dof025081	*DoWRKY66*		C2—H2	338	37433.44	9.93
Dof006469	*DoWRKY22*	Group IIe	C2—H2	365	40199.98	6.72
Dof027038	*DoWRKY73*		C2—H2	365	40158.93	6.72
Dof025778	*DoWRKY67*		C2—H2	340	37612.32	4.87
Dof004088	*DoWRKY17*		C2—H2	340	37638.30	4.87
Dof021212	*DoWRKY61*		C2—H2	344	38055.24	5.38
Dof011426	*DoWRKY38*		C2—H2	332	36572.59	4.69
Dof011427	*DoWRKY39*		C2—H2	339	37487.70	4.86
Dof000555	*DoWRKY4*		C2—H2	307	33640.45	5.06
Dof000256	*DoWRKY1*		C2—H2	281	30172.30	4.95
Dof009129	*DoWRKY31*		C2—H2	94	10575.26	9.69
Dof026085	*DoWRKY69*		C2—H2	353	38486.09	5.58
Dof027215	*DoWRKY74*	Group III	C2—HC	196	23160.74	7.01
Dof022978	*DoWRKY62*		C2—HC	444	51407.33	7.58
Dof022979	*DoWRKY63*		C2—HC	156	17488.65	8.45
Dof019216	*DoWRKY57*		C2—HC	106	11988.34	6.09
Dof019217	*DoWRKY58*		C2—HC	141	16047.09	8.75
Dof002691	*DoWRKY12*		C2—HC	229	25487.59	4.95
Dof002693	*DoWRKY13*		C2—HC	254	28058.35	6.16
Dof014289	*DoWRKY43*		C2—HC	238	27240.00	7.73
Dof007602	*DoWRKY28*		C2—HC	200	21651.19	5.50
Dof008579	*DoWRKY29*		C2—HC	295	33559.59	4.79
Dof016370	*DoWRKY48*		C2—HC	306	33962.10	5.44
Dof016367	*DoWRKY47*		C2—HC	436	47596.64	6.40
Dof011275	*DoWRKY35*		C2—HC	176	19844.98	5.85
Dof005880	*DoWRKY21*		C2—HC	294	33021.02	5.42
Dof019734	*DoWRKY59*		C2—HC	252	27576.94	5.12
Dof010267	*DoWRKY33*		C2—HC	1,583	181557.68	4.90
Dof003360	*DoWRKY15*		C2—HC	365	39231.78	5.83

### The phylogenetic analysis of *DoWRKY* genes

**FIGURE 2:**
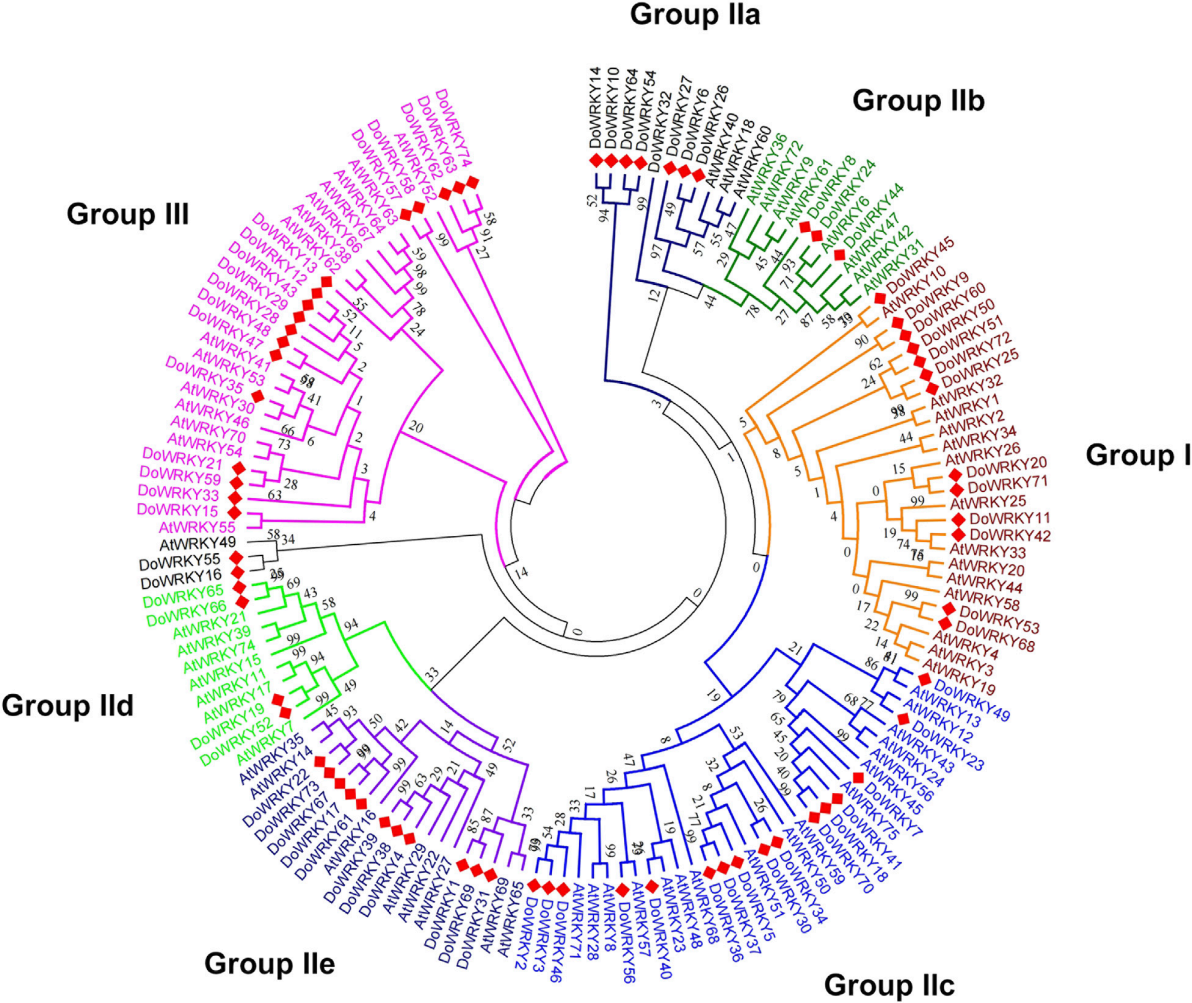
The phylogenetic tree of DoWRKYs and AtWRKYs by neighbor-joining method.

**FIGURE 3:**
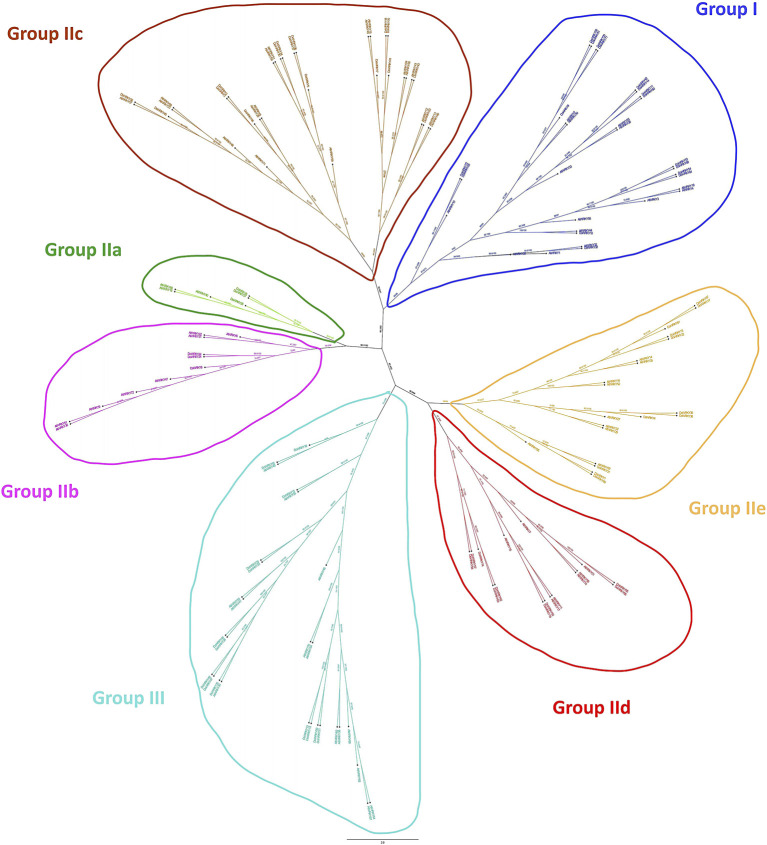
The phylogenetic tree of DoWRKYs and AtWRKYs by maximum likelihood method.

We imported the WRKY transcription factors into MEGA 6.0. The Dendrobium.fa file and the WRKY transcription factor aligned the muscular function of *A. thaliana* to compare and analyze the protein sequences of the two WRKY transcription factors, and we acquired the WRKY transcription factors of the two species after alignment. The NJ method was used to generate a phylogenetic tree map from sequence differences. An analogy was made to the related DoWRKY transcription factors based on the grouping of distinct WRKY transcription factors in the evolutionary tree, alluding to the obvious classification of AtWRKY transcription factors. In *Dendrobium*, WRKY transcription factors can be found in different parts of the plant, and the comparison and analysis of amino acids were fully described (Figure 2). The results indicated that all DoWRKYs could be divided into three groups and clustered separately from AtWRKYs. However, due to the distant relationship between *Arabidopsis* and *Dendrobium*, it can be seen that orthologs on the same branch, such as DoWRKY45 and AtWRKY10, DoWRKY49 and AtWRKY13, DoWRKY23 and AtWRKY12, DoWRKY35 and AtWRKY30, etc*.*, have no similarity greater than 80%. To more accurately display the degree of homology between DoWRKY and other AtWRKY10 members, we used IQ-TREE software to construct an ML tree (Figure 3). The results show that these DoWRKYs can still be dispersed into three groups and five subgroups (group II). The clustering of the phylogenetic tree constructed by the ML method was basically consistent with the evolutionary tree constructed by the NJ method. Interestingly, we found that DoWRKY from group IIc could be further divided into two branches; we temporarily call them group IIc-1 (left) and group IIc-2 (right). In group IIc-1 subclade, there are 10 DoWRKYs clustered in one branch, while only seven DoWRKYs were in group IIc-2 subclade.

### The conserved motif analysis of *DoWRKY* genes

**FIGURE 4:**
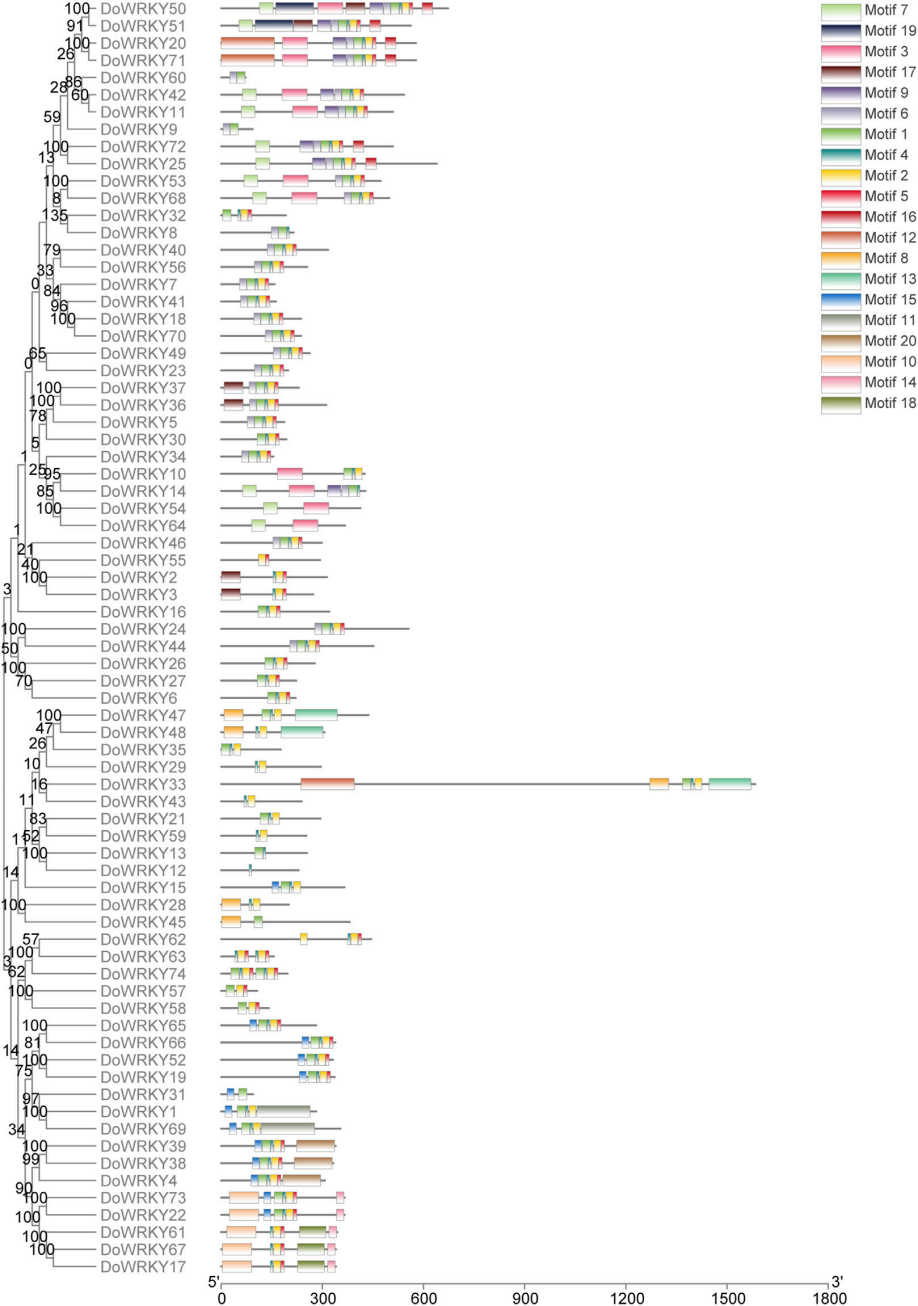
Analysis of the conserved motifs of *DoWRKY* genes.

To investigate the conserved motifs of *DoWRKY* genes, the screened DoWRKY protein sequence.fa file was submitted to the MEME online analysis website. Rom the xml files, the TBtools software was used to visualize motif patterns, set and retain 20 motifs, and obtain the map of conserved motif analysis. The analysis results show that the number and types of motif structures of different DoWRKY transcription factors are quite different, but the number and types of motif structures of the same type of *DoWRKY* genes are very close. According to the phylogenetic tree, the conserved motifs of *DoWRKY* genes were as follows: motif 1 and motif three are characteristic motifs containing WRKYGQK, which are in line with phylogenetic classification. The number of motifs contained in each WRKY transcription factor varies from 1 to 11, among which class I *DoWRKY* generally has the most motifs. Some *DoWRKY* genes, like motifs 9, 17, 19, etc*.*, have subfamily-specific motif sequences. The distribution of specific motif was shown in Figure 4.

### The gene structure analysis of *DoWRKY* genes

**FIGURE 5:**
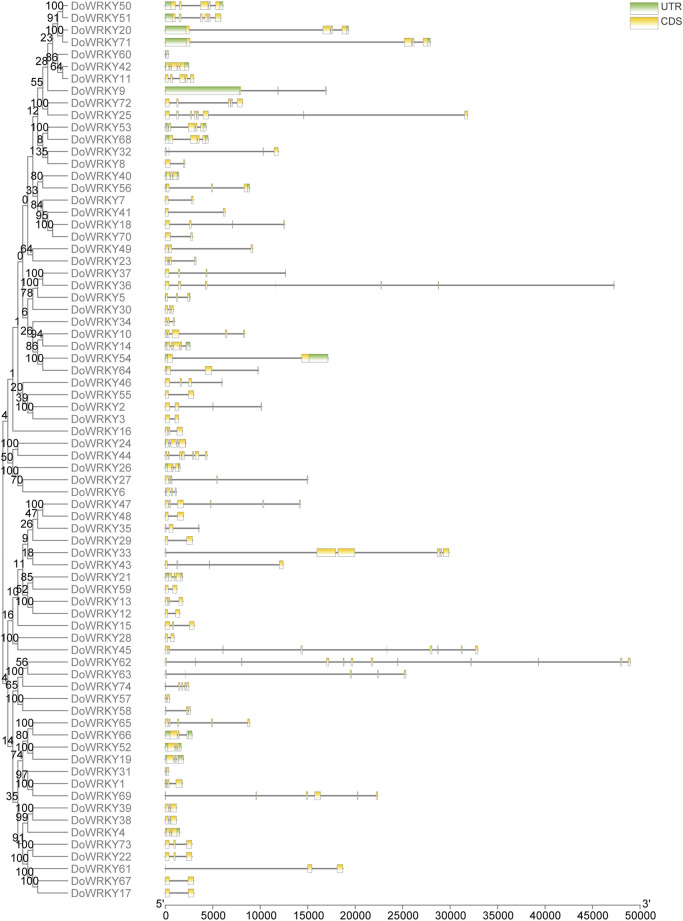
The gene structure analysis of *DoWRKY* genes.

The diversity of gene structures is favorable for the emergence of large gene families. The genomic structure of each *DoWRKY* gene was mapped. To better understand the gene structure, a phylogenetic tree was made based on the full-length DoWRKY proteins. Following the extraction of the gene ID corresponding to the screened DoWRKY transcription factors, the TBtools software was employed to visualize the gene structure based on the gene annotation file. Different types of *DoWRKY* genes vary greatly in length, numbers of exons and introns, and starting sites. All *DoWRKY* genes have 1-6 exons. WRKY-like transcription factor genes have the largest number of exons. The number of introns in *DoWRKYs* is between 0 and 3, and most of them have 1-2 introns. The same type of gene structure in *DoWRKYs* was very similar. Most of them have the same number of exons. CDS start sites and UTR were also very close. The distribution of exons and introns is very different between different subclasses, such as class I and class III. There are differences in gene length between classes, and large differences in the distribution of exons and introns (Figure 5).

### The chromosomal location of *DoWRKY* genes

**FIGURE 6:**
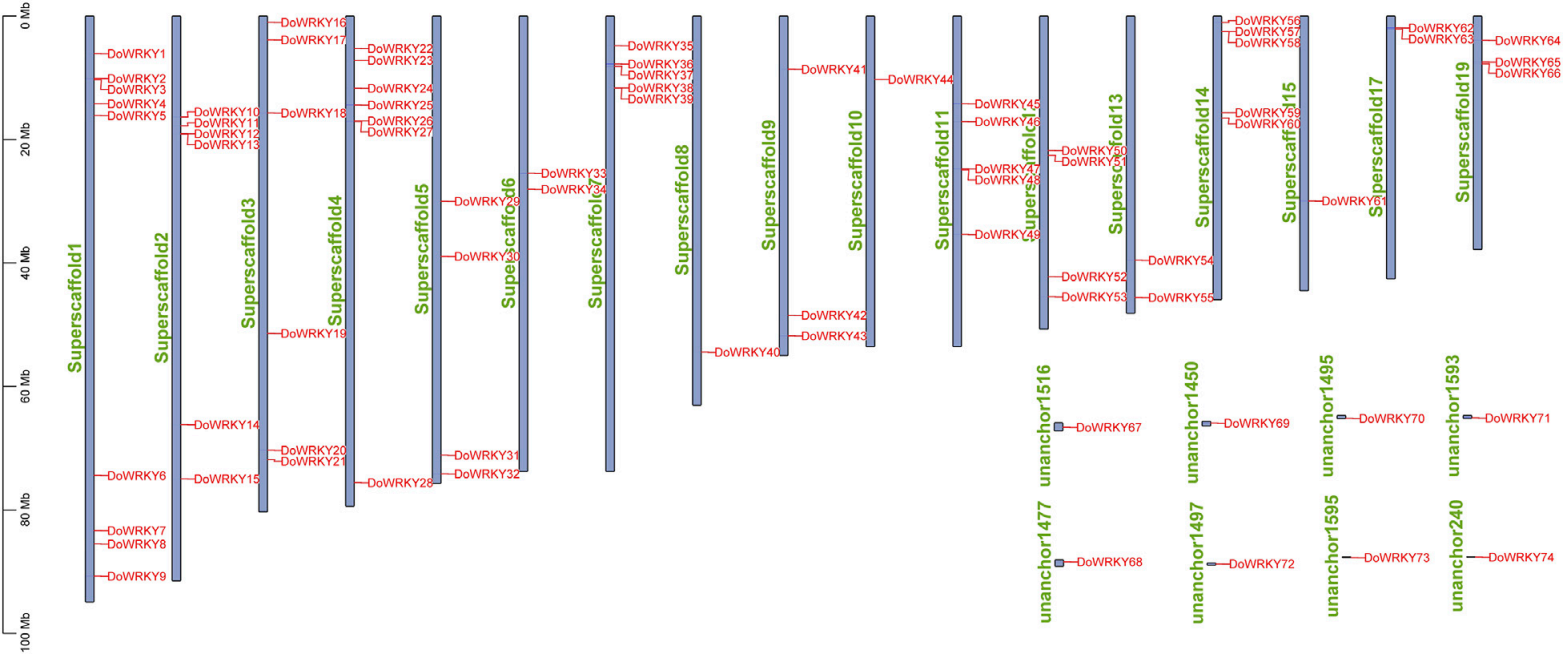
The chromosome location analysis of *DoWRKY* genes.

From the Dendrobium genome annotation information gff3 file, TBtools software was used to perform the chromosomal location analysis based on the gene annotation file. After gene renaming, those genes can be introduced into the file to obtain the location of the WRKY transcription factor gene on the genome. It is found that 74 *DoWRKY* genes are irregular and uneven. The distribution of *DoWRKY* genes on each chromosome segment, some of which are not located on the chromosome, may be caused by the fact that these *DoWRKY* genes are not annotated on the chromosome. The chromosomal location of the gene was shown in Figure 6. As *DoWRKY* genes were unevenly distributed throughout all chromosomes, and the number of genes on each chromosome was unrelated to its length. Eight genes, including *DoWRKY67*, *DoWRKY69*, *DoWRKY70*, etc*.*, were dispersed throughout eight large segments while the majority of genes were distributed across 19 scaffolds. Chromosome 1 contained the largest number of *DoWRKY* genes. Several patterns of gene duplication have been observed, such as whole-genome duplication (or segmental duplication) and tandem duplication. The genes from tandem duplication, including *DoWRKY2* and *DoWRKY3*, *DoWRKY12* and *DoWRKY13*, *DoWRKY26* and *DoWRKY27*, *DoWRKY38* and *DoWRKY39*, etc*.*, are scattered throughout the genome. Those genes with higher homology but not located on the same chromosome may be attributed to segmental duplication.

### The expression profile of *DoWRKY* genes under MeJA treatment

**FIGURE 7:**
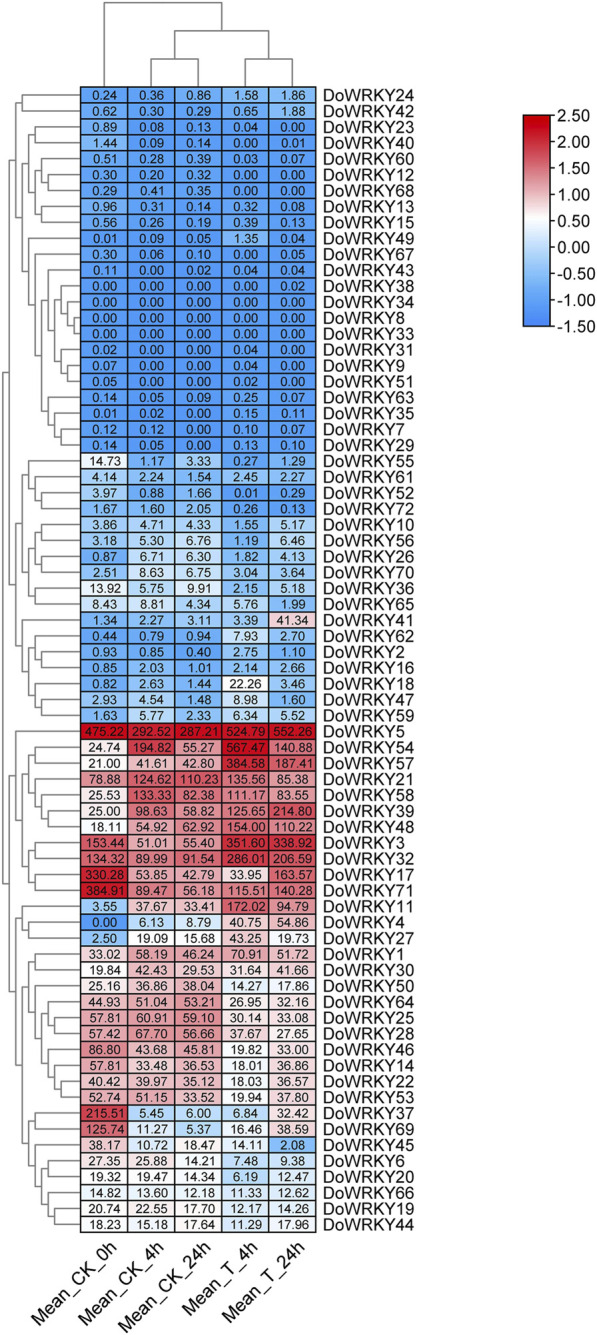
The expression profiles of *DoWRKYs* after MeJA treatment. The expression level of genes were normalized by log2 (FPKM value).

To further clarify the roles of the *WRKY* genes in abiotic stress, we conducted a comparative transcriptomic analysis of MeJA-treated *D. officinale* at different stages (Jiao et al., 2022). The results showed that WRKY family members were differentially expressed at different treatment times and showed two expression patterns (Figure 7). The first is that most *WRKY* genes are not expressed before and after treatment, and a small number of WRKY genes are down-regulated; the second is that this group of *WRKY* genes is significantly up-regulated, especially *DoWRKY5*. Whether in the control or MeJA treatment groups, a portion of the *WRKY* genes, such as *DoWRKY54*, *57*, *21*, and *58* reached the highest expression level at 4 h and gradually decreased at 24 h but did not return to the initial level (Supplementary Table S2). Therefore, the latter may be involved in the regulation of JA-responsive genes. The relevant transcriptional regulation mechanism needs further validation.

## Discussion

**FIGURE 8:**
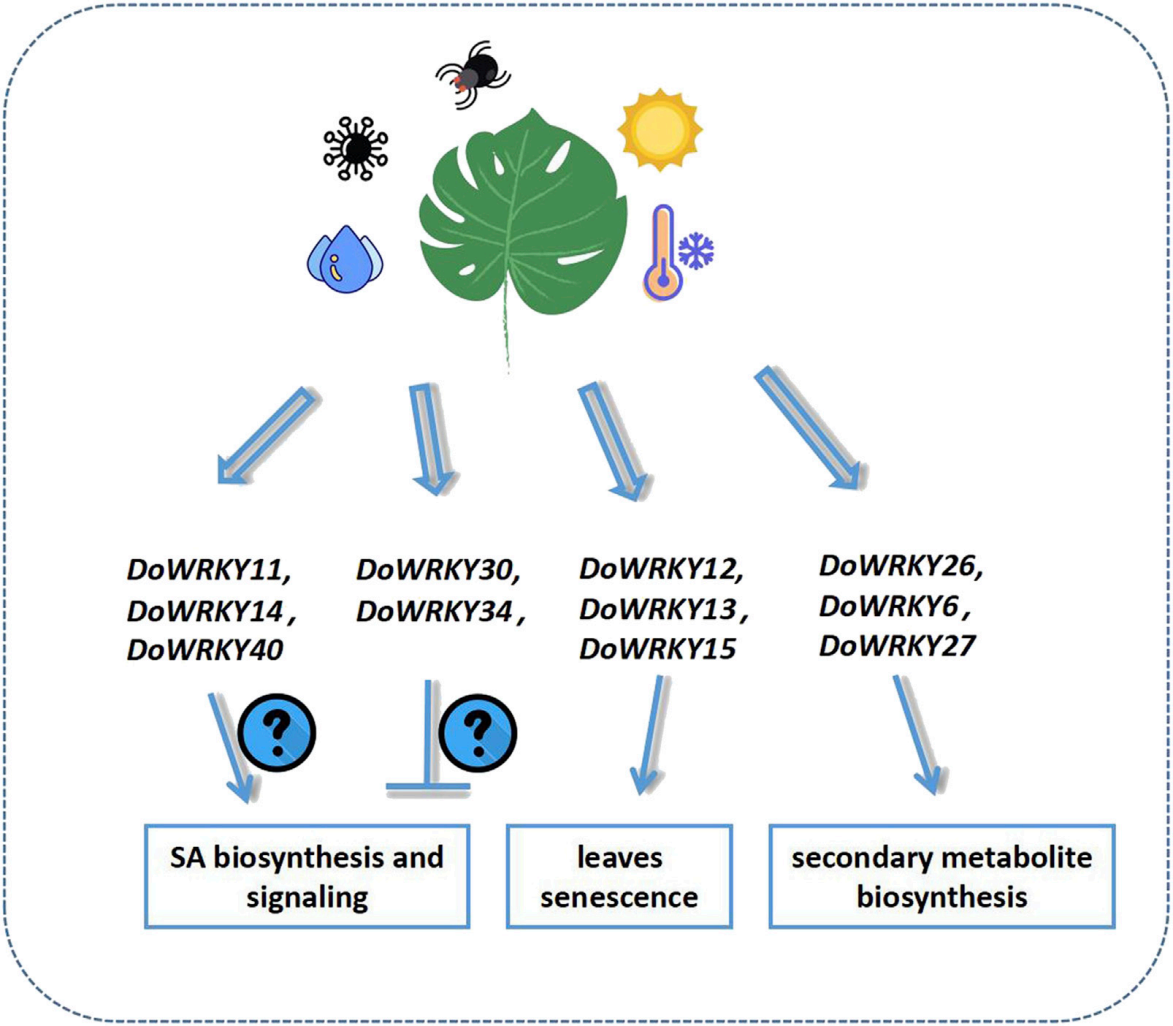
The putative roles of some *DoWRKYs* in *D. officinale*.

As a crucial family of transcription factors in the regulation of plant growth, development, and stress resistance, WRKY transcription factors are involved in a variety of physiological processes in plants. The genome-wide investigation of the WRKY gene family has been undertaken thoroughly in hundreds of species, and the genomes of many species have begun to be sequenced gradually (Li et al., 2019). Owing to the achievement of cutting-edge high-throughput sequencing technology, some Dendrobium species have been widely sequenced in the last 5 years. In this study, the WRKY genes in the *Dendrobium* genome were identified, and 74 of them met the criteria. This was less than the 91 WRKY transcription factors found in the *Arabidopsis* genome. The majority of them shared similar domains with AtWRKYs, and they all have the WRKYGQK and zinc finger structures with ZF. The heptapeptide sequences of DoWRKY include WTKTGQK, WKKYGQK, WRKYGRD, WRKYGKK, and other types. The group II WRKY transcription factors in Dendrobium have a maximum of 44 members, whereas groups I and III merely have 13 and 17 members, respectively. The phylogenetic analysis indicated that groups I and III of Dendrobium are more closely related than groups II-a and II-b. The same branch is closely connected, and II-d and II-e are likewise related. The conserved domains showed that *DoWRKY* genes contain multiple conserved domains. There are additional subgroups of typical conserved domains in addition to the core WRKUGQK conserved domains. The conserved domains of DoWRKY are mostly similar. The group I DoWRKYs have the most conserved domains and may have significant regulatory functions (He et al., 2017; Tang et al., 2021). In Arabidopsis, the proteins AtWRKY25 and AtWRKY23 may interact with MAP kinase four substrate 1 (MKS1), which is essential for the regulation of SA-dependent resistance. The SA-related defense gene *PR1* was identified to be expressed at a higher level in an *Atwrky33* mutant (Andreasson et al., 2005). AtWRKY50 and AtWRKY51 proteins controled both SA- and low oleic acid-dependent inhibition of JA signaling, resulting in greater resistance to *Alternaria brassicicola* but increased susceptibility to *Botrytis cinerea* (Gao et al., 2011). AtWRKY57 regulates the plant immune response process and competes with AtWRKY33 by binding to the promoters of JAZ1 and JAZ5 (Jiang and Yu 2016). DoWRKY11 and DoWRKY14 clustered with AtWRKY25 and 33 in one clade, while DoWRKY40 clustered with AtWRKY23 in another subclade. DoWRKY30, DoWRKY34, and AtWRKY50/51 merged a single branch. These results imply that DoWRKY11, 14, 40 and DoWRKY30, 34 have different regulatory effects on the target genes involved in SA signaling. In addition to being involved in phytohormone signaling, WRKY genes are involved in leaf senescence. AtWRKY22, AtWRKY54, AtWRKY70, AtWRKY57, AtWRKY45, AtWRKY75, AtWRKY6, AtWRKY46, AtWRKY25, AtWRKY53, and AtWRKY55 participate in the progression of leaf senescence. AtWRKY54, 70, has high homology with DoWRKY12, 13, and DoWRKY21, 59, respectively. DoWRKY15 and AtWRKY55 were clustered into one branch, which suggested that they might be involved in the leaves senescence process of *D. officinale*. Many studies had also shown that some members of the WRKY family helped mediate the biosynthesis of secondary metabolites in plants (Schluttenhofer and Yuan, 2015). *D. officinale* is also an important Chinese herbal medicine. It will be of great significance if some key *DoWRKY* genes can be isolated. *AtWRKY18* and *AtWRKY40* are implicated in camalexin and indole-glucosinolate biosynthesis, and the accumulation of these compounds was required for resistance toward Golovinomyces orontii (Schön et al., 2013). The ectopic expression of AtWRKY18, AtWRK40, and AtMYC2 activated the MEP pathway genes of Salvia sclarea, leading to an increase in abietane diterpene levels (Alfieri et al., 2018). In this study, DoWRKY26 shared a high level of homology with AtWRKY18, 40, and 60; hence, it was suggested that WRKY26 might be involved in the transcriptional regulation of secondary metabolite biosynthetic genes (Figure 8).

Gene duplications are critical to the rapid expansion of the genome and the evolution of gene families (Song et al., 2022c). Polyploidy drives species adaption, genetic diversity, and genome evolution. In the field of horticulture, chromosomal polyploidy produces a number of excellent orchid varieties (Vilcherrez-Atoche et al., 2022). Much evidence indicated that duplication and expansion of *WRKY* genes occurred in the interspecies (Yang et al., 2018). Numerous evidence suggested that duplication and expansion of WRKY genes occurred among species (Yang et al., 2018). The sequencing of the whole genome of *A. shenzhenica* boosts our knowledge of the history and evolution of those subfamilies (Zhang et al., 2017). It is notable that whole genome duplication (WGD) has occurred more than once in plant genomes (Clark and Donoghue, 2018). Large-scale tandem duplication or segmental duplication start driving the generation of new genes and speciation evolution (Clark and Donoghue, 2018). Typically, orchids have experienced WGD repetitively, including a historical WGD event and a recent WGD event shared by all orchids. Mycoheterotrophic and parasitic orchids exist with the great majority of ornamental orchids. The loss and survival of symbiotic genes connected to the evolution of particular symbionts spans from the ancestral arbuscular mycorrhiza to the recent ericoid and orchid mycorrhizae (Barrett et al., 2019; Gao et al., 2020). Here, nine tandem duplication gene pairs of *DoWRKY* were confirmed in peusodochromosomes. Those distributed across multiple scaffolds with a high degree of similarity may result in segmental duplications. This observation is consistent with that of several model plants. The gene structure of *DoWRKYs* within the same family is comparable, as are the number of CDS and initiation sites, as well as the number and location of introns, despite the major differences within families, such as group I. The number of CDSs and intron sites differs significantly between group I and III, implying that class I WRKY transcription factors have more functions. *DoWRKYs* are unevenly located on different chromosomes or segments, and some have not yet been located on the chromosome, such as *DoWRKY54/59*.

## Conclusion

The WRKY transcription factor family, as one of the largest TF families, plays a significant and necessary role in plants. Over the years, it has been demonstrated that WRKYs not only contribute to plant growth and development but also exhibit intricate regulatory mechanisms. Here, the identification of *DoWRKY* reveals the membership of the *DoWRKY* gene family and elucidates the function and structure of each gene, which could provide a theoretical basis for future functional verification studies. Using the homologous gene alignment of several species, it is possible to simultaneously identify similar homologous genes with great affinity for their motifs as well as infer the potential functions of related subgroup genes. We combined TBtools, MEGA, and other software to identify 74 *DoWRKY* genes, along with the analysis of amino acid alignment, gene structure, conserved motif, phylogeny, and chromosomal location. Large-scale gene duplication may contribute to the functional preference of the *WRKY* genes. This includes the emergence of tandem replicated genes. By analyzing conserved motifs and domains, we can divide *WRKY* genes into three groups. Analysis of phylogenetic tree indicated that some *DoWRKY* genes shared homology with AtWRKY, which allowed us to speculate on their potential functions. In addition, we preliminarily deduced that *DoWRKY5, 54*, *57*, and *21* may be involved in the JA signaling by analyzing the expression patterns. As a result, we can learn more about WRKY transcription factors and how they work, and we can also use the results of this analysis to learn more about WRKY genes and their functions in other species.

## Data Availability

The datasets presented in this study can be found in online repositories. The names of the repository/repositories and accession number(s) can be found in the article/Supplementary Material.
